# Strain-Dependent Migration of CD4 and CD8 Lymphocyte Subsets to Lymph Nodes in NOD (Nonobese Diabetic) and Control Mice

**DOI:** 10.1155/1994/32820

**Published:** 1994

**Authors:** Christelle Faveeuw, Marie-Claude Gagnerault, Françoise Lepault

**Affiliations:** CNRS URA 1461 Hôpital Necker Paris 75015 France

**Keywords:** Strain-dependency, homing, lymph nodes, CD4, CD8, NOD

## Abstract

Subpopulations of lymphoid cells were compared with respect to their ability to migrate into peripheral lymphoid organs of nonobese diabetic (NOD) mice and various strains of control mice. In short-term, *in vivo* homing studies, no major differences in the pattern of homing of B and T cells were observed among all mouse strains studied. On the other hand, CD4 cells localized consistently more efficiently than CD8 cells in both PP and LN of adult NOD and BALB/c mice, whereas both populations migrated roughly equivalently in LN of adult DBA/2, CBA, and C57BL/6 mice. No age-dependent differences in the homing of CD4 and CD8 cells were observed in BALB/c mice. On the contrary, in 2-week-old NOD mice, CD4 and CD8 cells migrated equally well. The preferential entry of CD4 cells in adult NOD and BALB/c did not result from increased blood transit time of CD8 cells. On the other hand, the preferential migration of CD8 cells was observed in the liver, whereas the two T-cell subsets migrated equally well in the lungs. The differences in the homing characteristics of CD4 and CD8 cells among NOD, BALB/c, and C57BL/6 mice were not related to modifications in the level of expression of adhesion molecules such as MEL-14, LFA-1, and Pgp-1.


*Developmental Immunology*, 1994, Vol 3, pp. 273–282
Reprints available directly from the publisher
Photocopying permitted by license only

© 1994 Harwood Academic Publishers GmbH
Printed in Singapore

### Strain-Dependent Migration of CD4 and CD8 Lymphocyte Subsets to Lymph Nodes in NOD (Nonobese Diabetic) and Control Mice

CHRISTELLE FAVEEUW,*† MARIE-CLAUDE GAGNERAULT,† and FRANÇOISE LEPAULT†

†*CNRS URA 1461, Hôpital Necker, 75015 Paris, France*

Subpopulations of lymphoid cells were compared with respect to their ability to migrate into peripheral lymphoid organs of nonobese diabetic (NOD) mice and various strains of control mice. In short-term, *in vivo* homing studies, no major differences in the pattern of homing of B and T cells were observed among all mouse strains studied. On the other hand, CD4 cells localized consistently more efficiently than CD8 cells in both PP and LN of adult NOD and BALB/c mice, whereas both populations migrated roughly equivalently in LN of adult DBA/2, CBA, and C57BL/6 mice. No age-dependent differences in the homing of CD4 and CD8 cells were observed in BALB/c mice. On the contrary, in 2-week-old NOD mice, CD4 and CD8 cells migrated equally well. The preferential entry of CD4 cells in adult NOD and BALB/c did not result from increased blood transit time of CD8 cells. On the other hand, the preferential migration of CD8 cells was observed in the liver, whereas the two T-cell subsets migrated equally well in the lungs. The differences in the homing characteristics of CD4 and CD8 cells among NOD, BALB/c, and C57BL/6 mice were not related to modifications in the level of expression of adhesion molecules such as MEL-14, LFA-1, and Pgp-1.

KEYWORDS: Strain-dependency, homing, lymph nodes, CD4, CD8, NOD.

### INTRODUCTION

Mature lymphocytes migrate continuously among lymphoid organs via the blood and lymph vessels. Lymphocytes leave the blood by selectively adhering, binding to, and migrating through postcapillary high endothelium venules (HEV) in lymphoid organs. Two distinct lymphocyte-HEV recognition systems control homing of lymphocytes into lymphoid organs, one for peripheral lymph nodes (pLN) and one for mucosal lymphoid organs (Butcher et al., 1980). Complementary molecules expressed on both the T-cell surface and the endothelium mediate tissue-specific endothelial-cell recognition. The first lymphocyte homing receptor identified is defined by the MEL-14 antibody, which blocks homing of lymphocytes into pLN (Gallatin et al., 1983). Binding of mouse lymphocytes to Peyer's patch (PP) HEV is at least partly mediated by the integrins $\alpha 4\beta 1/\alpha 4\beta 7$ (LPAM-1/2) (Holzmann et al., 1989; Holzmann and Weissman, 1989). Other lymphocyte receptors involved in HEV recognition, such as the integrin LFA-1 or the molecule Pgp-1, are not organ-specific (Jalkanen et al., 1987; Hamann et al., 1988; Picker et al., 1989). Endothelial-cell-surface molecules involved in tissue-specific interactions with lymphocytes include addressins, Ig family molecules, and integrins (Springer, 1990; Butcher, 1991; Shimisu et al., 1992).

The differential expression of receptors/counter receptors regulates migration patterns of different lymphoid populations and determines their representation in particular regions. Such mechanisms are involved in the establishment of inflammation. HEV-like venules expressing vascular addressins are observed in target organs of autoimmune diseases associated with lymphocytic infiltration such as rheumatoid arthritis (Jalkanen et al., 1986; Dinther-Janssen et al., 1990), EAE (Cannella et al., 1991), and insulin-dependent diabetes mellitus (Berg et al., 1989). The NOD mouse is an experimental model for human autoimmune type 1 diabetes, in which

*Corresponding author.

lymphocyte infiltration around and into the islets of Langerhans develops from 3–5 weeks of age in both sexes, whereas overt diabetes occurs predominantly in females from 12 weeks of age (Makino et al., 1980). The central role of T cells in insulin-secreting beta-cell destruction has been widely documented (Miyazaki et al., 1985; Ogawa et al., 1985; Mori et al., 1986; Schizuru et al., 1988; Bendelac et al., 1987; Sempé et al., 1991). The mononuclear cell infiltrate is mostly composed of T cells with a minority of B lymphocytes and macrophages (Miyazaki et al., 1985). The CD4/CD8 ratio in the infiltrate is clearly increased compared to peripheral lymphoid organs. This observation may suggest that in NOD mice, the T-cell subpopulations that enter the pancreas have migratory properties different from those of the bulk of recirculating lymphocytes. On the other hand, it has not yet been determined whether migratory properties of T-cell subsets in NOD are comparable to that of other mouse strains. To address this question, we examined the short-term homing of lymphocyte subpopulations into the peripheral lymphoid organs of NOD and several control mouse strains. We show that, in NOD as well as in BALB/c adult mice, CD4 cells home better into both PP and LN than do CD8 cells. The preferential migration of CD4 cells is even more pronounced in NOD than in BALB/c mice and is age-dependent in NOD but not in BALB/c mice. On the contrary, in several other mouse strains (CBA, C57BL/6, DBA/2), these two subsets home to LN equally well. These results demonstrate strain-dependent differences in the migratory properties of CD4 and CD8 subsets in LN in the mouse, whereas the preferential migration of CD4 and CD8 cells into PP is a general trait of all mouse strains. The data also suggest that any differences in homing of NOD lymphocytes are rather related to the mouse strain than to the autoimmune disease process.

### RESULTS

### Homing of $^{51}$Cr-Labeled Lymphocytes

Transfer of $^{51}$Cr-labeled lymphocytes from 10-week-old BALB/c and NOD females into sex- and age-matched syngeneic recipients showed no strain-dependent differences in the distribution of lymphocytes in the following organs: spleen, pLN, mLN, paLN, PP, intestine, liver, brain, kidneys, thymus, and bone marrow. Two hours after injection, 74.9 ± 5.6% of the total radioactivity was recovered in BALB/c mice and 63.1 ± 1.2% in NOD mice. Most of the lymphocytes were recovered from the spleen, liver, and lungs. Similarly, the migration of lymphocytes in organs such as the pancreas, salivary glands, lungs, and thyroid, which exhibit lymphocyte infiltration in adult NOD mice (Tochino, 1987), was not different in the two mouse strains (data not shown).

### B- and T-cell Localization

Experiments were conducted with NOD of both sexes and with C57BL/6 and BALB/c females at 10 weeks of age. The proportion of newly homed B and T cells in the spleen, pLN, mLN, paLN, and PP was determined in the three mouse strains. Because no differences were observed between male and female NOD mice, the results were pooled. No major differences in the homing characteristics of B and T cells of pLN, mLN were found between NOD and control mice (data not shown). As already shown for some mouse strains and for other species, B cells were more apt to enter the spleen and PP than LN, and total T cells migrated with the same efficiency into mLN, pLN, and PP (Stevens et al., 1982; Pals et al., 1986; Westermann et al., 1989).

### CD4 and CD8 T-Cell Subset Localization

The results, presented as relative migration indexes (RMI) in five different strains, show strain-dependent migration patterns of CD4 and CD8 subsets (Fig. 1). The preferential migration of CD4 cells into LN and PP was striking in NOD and BALB/c mice, where the RMI was 3 or greater. The difference in the migration rate of CD4 and CD8 lymphocytes was even greater in NOD than in BALB/c mice. Interestingly, in these two mouse strains, CD4 and CD8 cells migrated differentially into the spleen. The two subsets homed with the same efficiency in BALB/c mice (RMI = 1), whereas in NOD mice, CD4 cells migrated significantly better than CD8 cells. In C57BL/6, CBA, and DBA/2 mice, CD4 and CD8 cells migrated almost equally well into peripheral lymphoid organs, especially in the case of mLN. The RMI in peripheral lymphoid organs were significantly different between NOD and C57BL/6 and between BALB/c and C57BL/6 (except for PP).

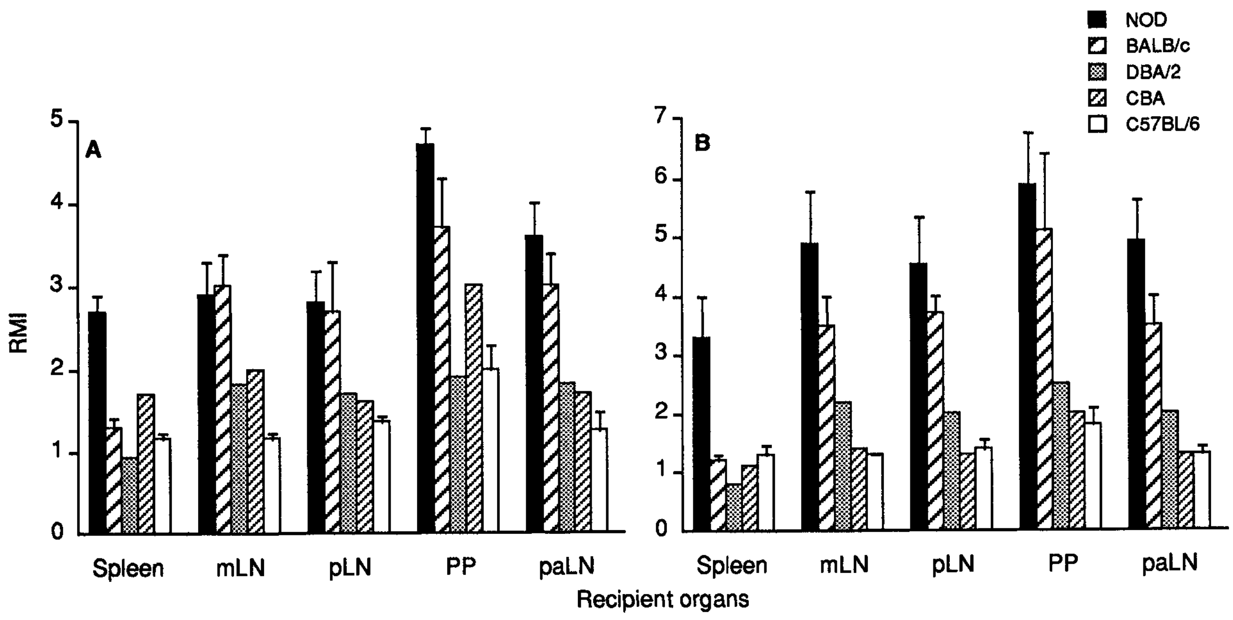

FIGURE 1. Differential localization of CD4 and CD8 subsets in secondary lymphoid organs. 2.5 × $10^7$ FITC-labeled cells from (A) pLN and (B) mLN of different adult strains of mice were injected i.v. into adult syngeneic recipients. An aliquot of the injected cells was kept to determine the CD4/CD8 ratio. Two hours later, cell suspensions of the recipients' pLN, mLN, spleen, PP, and paLN were prepared. Organs were pooled from three recipients. The percentages of donor-type CD4 and CD8 cells were determined by immunofluorescence in each recipient organ. 500 FITC-labeled cells were scored using a FACScan. The results are expressed as RMI. They represent the mean ± SE of two, three, and four experiments for C57BL/6, BALB/c, and NOD mice, respectively. For DBA/2 and CBA mice, the histograms represent data from one experiment.

### CD4 and CD8 T-Cell Subset Localization as a Function of Age

In the NOD mouse, insulitis starts around 3–4 weeks of age. To determine whether there is a correlation between the pattern of migration of lymphocytes and the severity of insulitis, homing properties of CD4 and CD8 lymphocytes were compared in 2-, 10-, and 20-week-old NOD mice and in recently diabetic animals (Fig. 2). No age-dependent differences were observed in the proportions of donor B and total T cells in age-matched recipients' organs (data not shown). In NOD mice aged 10 weeks or older and in recently diabetic mice, CD4 cells migrated significantly better than CD8 cells to LN. On the contrary, in young NOD animals (2 weeks), CD4 and CD8 cells migrated equally well. In the spleen of NOD mice, the migration of CD4 and CD8 lymphocytes was independent of the age of the mouse. In BALB/c mice, no significant age-dependent differences in the homing of the two T-cell subsets into peripheral lymphoid organs were observed.

### Donor and Recipient CD4 and CD8 Subsets in the Blood

The different migratory properties of CD4 and CD8 cells in NOD and BALB/c mice could result in an increased blood transit time of CD8 cells as compared to CD4 cells. Therefore, the CD4/CD8 ratio of the FITC-labeled cells that remained in the blood stream 2 hr after injection was calculated (Table 1). The results show that this ratio was not different from that of the injected cells in adult BALB/c, and

TABLE 1
Differential Distribution of Donor CD4 and CD8 Subsets in the Blood of Recipients

| Strain | Age (weeks) | CD4/CD8 ratio | |
|---|---|---|---|
| | | Injected LN cells | Blood borne LN cells |
| NOD | 2 | 2.8 ± 0.1[a] | 3.2[b] |
| | 10 | 2.7 ± 0.2 | 4.4 |
| BALB/c | 10 | 2.5 ± 0.2 | 2.8 |

[a] Each result is the mean ± SE of three to eight animals.

[b] The CD4/CD8 ratio was determined 2 hr after injection of FITC-labeled LN cells. Each value represents the mean of two animals.

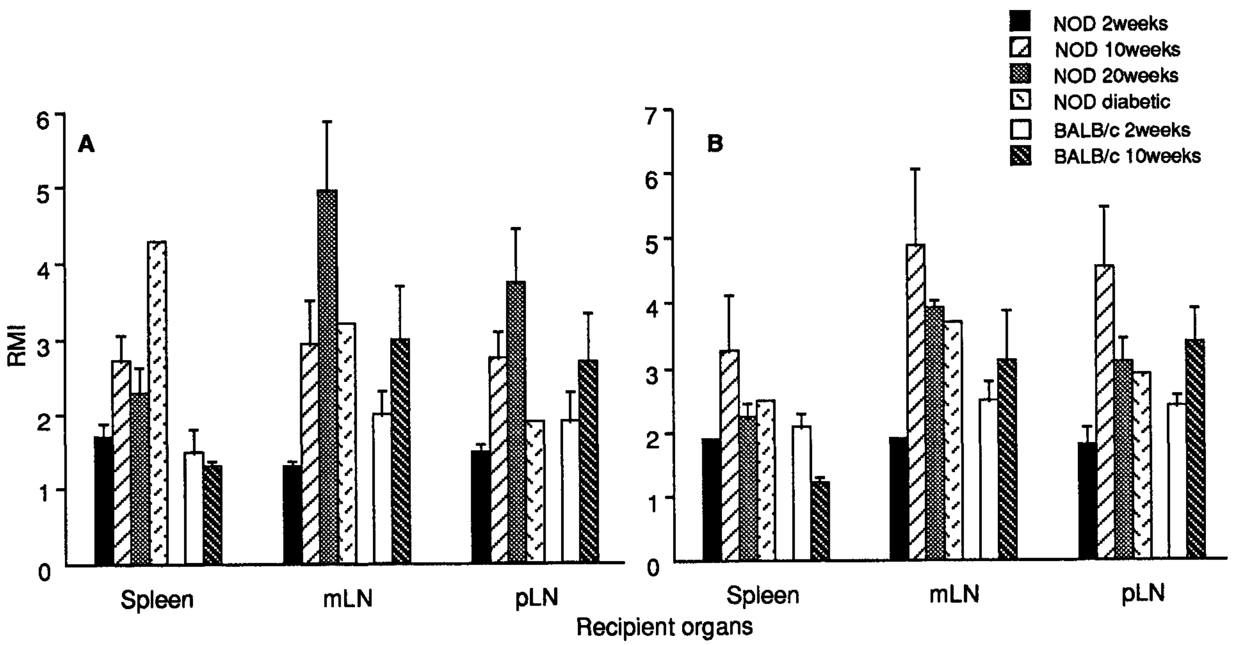

FIGURE 2. Age-related variation of the RMI in the peripheral lymphoid organs of NOD and BALB/c mice. (A) pLN and (B) mLN FITC-labeled cells from NOD and BALB/c mice of different ages were injected into syngeneic recipients of the same age. Two hours later, cell suspensions of the recipients' spleen, pLN, and mLN were prepared. Organs were pooled from three adult or five young recipients. Data presentation is as described for Fig. 1. The results represent pooled data from three to four experiments.

in 2- and 10-week-old NOD mice. Thus, CD4 and CD8 cells are extracted from the blood at the same rate, suggesting that CD8 cells migrate to other parts of the body.

### CD4- and CD8-cell Migration in the Liver and the Lungs of Adult NOD Mice

Because the homing of lymphocytes into the liver and the lungs occurred at a high rate (data not shown), we examined the *in vivo* distribution of CD4 and CD8 cells in these two organs. As shown in Table 2, the RMI in the liver was below 1, indicating a preferential homing of CD8 cells in the liver, whereas in the lungs, CD4 and CD8 cells migrated equally well. These patterns of migration

TABLE 2
Distribution of Donor and Recipient CD4 and CD8 Cells in the Lungs and the Liver of Adult NOD Mice

| Organs | RMI[a] | CD4/CD8 *in situ*[b] |
|---|---|---|
| Liver | 0.5 ± 0.1 | 2.0 |
| Lungs | 1.0 ± 0.2 | 3.5 |

[a] RMI determined 2 hr after injection of LN FITC-labeled cells. Each result is the mean ± SE of three animals.

[b] Each value represents the mean of two animals.

did not reflect the *in situ* representation of the CD4 and CD8 lymphocytes in these two organs, where the CD4 phenotype predominates (Table 2).

### CD4- and CD8-cell Migration in the NOD Mouse as a Function of Time

The pLN cell suspensions from Thy-1.2 donors were injected into Thy-1.1 recipients. The mice were killed at intervals during 8 weeks and the organs removed for cell staining. The total T-cell population in the inoculum comprised 75% CD4 and 25% CD8 cells. If the lymphocyte subsets migrated equally well into the different organs, the percentage of each subset among migrating cells and in the inoculum should have been similar (Fig. 3). As already mentioned, 2 hr after injection, the percentage of donor type CD4 cells was higher and that of CD8 cells was lower than in the injected population, resulting in an increased CD4/CD8 ratio. Thus, the differential migration of T-cell subsets is observed whether the injected cells are labeled with FITC or express a different Thy-1 allele. Such a migration profile lasted for roughly 3–4 weeks in pLN and mLN. Later, the percentage of immigrant CD4 cells became equal to that of CD4 cells in the inoculum.

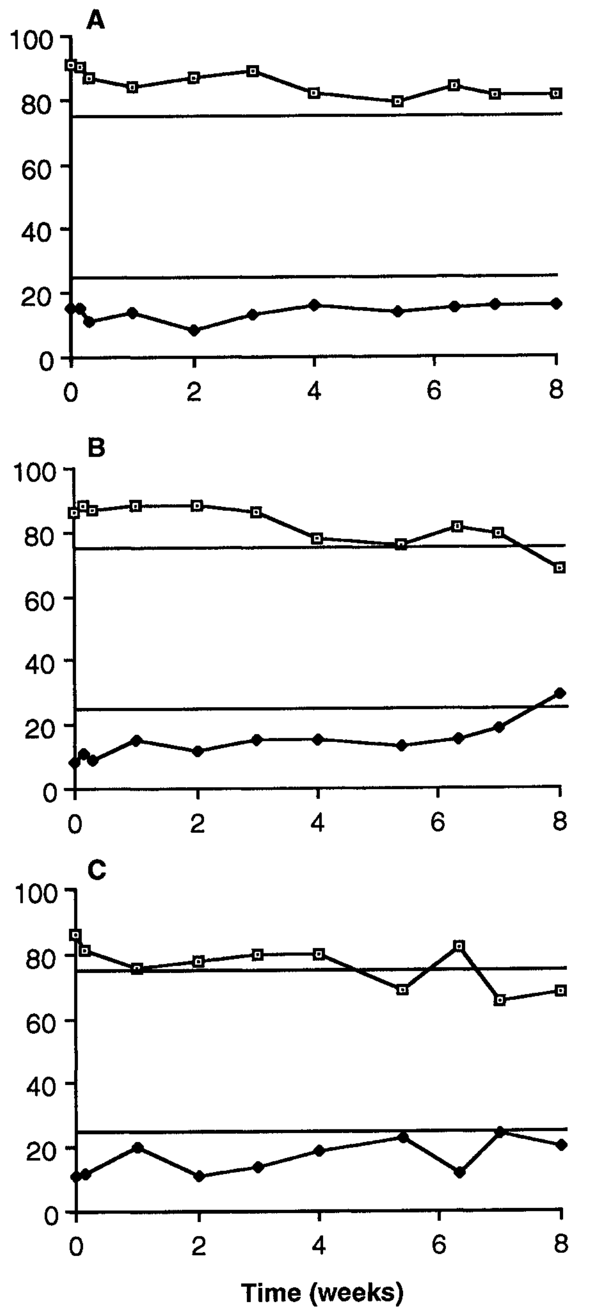

FIGURE 3. Time-course localization of CD4 and CD8 subsets in different organs of adult NOD mice. The pLN cells from Thy-1.2 donors were injected i.v. into Thy-1.1 recipients. Each recipient received $2.5 \times 10^7$ cells. At various time points as indicated, cell suspensions of (A) pLN, (B) mLN, and (C) PP were prepared. Organs were pooled from two recipients. The percentage of the donor-type CD4 (open squares) and CD8 (closed diamonds) subsets were determined in each recipient organ by double immunofluorescence staining, and compared to the percentage of CD4 and CD8 cells in the inoculum (75% and 25%, respectively) (straight lines). The results are expressed as the percentage of CD4 and CD8 cells among the Thy-1.2 donor lymphocytes recovered in recipient organs.

Conversely, the fraction of immigrant CD8 cells remained below the percentage of CD8 cells in the inoculum for 7 weeks in mLN and longer in pLN. In the PP, after 1 week, the percentage of donor-type CD4 cells decreased and was comparable to that of the inoculum, whereas the percentage of immigrant CD8 cells remained low for 4 weeks.

### Expression of MEL-14 Antigen, LFA-1, and Pgp-1 Adhesion Molecules in the Periphery

Because differences in the migratory properties of CD4 and CD8 cells in NOD and BALB/c mice (as compared to C57BL/6 mice) could result from modifications in the expression of adhesion molecules between these two T-cell subsets, we studied the expression of MEL-14 antigen, LFA-1, and Pgp-1 molecules known to be involved in the homing of lymphocytes in the periphery. Seventy-five to 90% of CD4 and CD8 cells expressed MEL-14 antigen, and 95 to 99% of the cells were LFA-$1^+$ in the three mouse strains. The proportion of MEL-14+ cells was slightly higher in BALB/c and C57BL/6 mice compared to NOD mice. As previously described (Ohgama and Onoé, 1992), CD8 lymphocytes expressed a higher level of the MEL-14 antigen and similar results were observed for LFA-1 expression. Moreover, a higher level of expression of LFA-1 on the two T-cell subsets in C57BL/6 as compared to NOD and BALB/c mice was observed. No age-dependent differences were observed in the expression of MEL-14 antigen and LFA-1 (data not shown). Figure 4 shows the expression of Pgp-1 on CD4 and CD8 LN cells in the three mouse strains aged 10 weeks. The expression of Pgp-1 on the two T-cell subsets was higher in BALB/c than in C57BL/6 mice and intermediate in NOD mice at all ages. The same profile was observed in young and old animals. In 3-week-old mice, 30% of CD4 and CD8 cells expressed high levels of Pgp-1 (Pgp-$1^{hi}$) in BALB/c mice, whereas in C57BL/6 mice, 11% of CD4 and 19% of CD8 cells were Pgp-$1^{hi}$. In young NOD mice, 24% of CD4 and 21% of CD8 cells were Pgp-$1^{hi}$. As already reported (Budd et al., 1987), there was a decrease in the Pgp-1 population in the oldest animals of all strains (data not shown). Overall, the different migratory properties of CD4 and CD8 cells in BALB/c, NOD, and C57BL/6 mice most likely do not result from differential expression of these adhesion molecules.

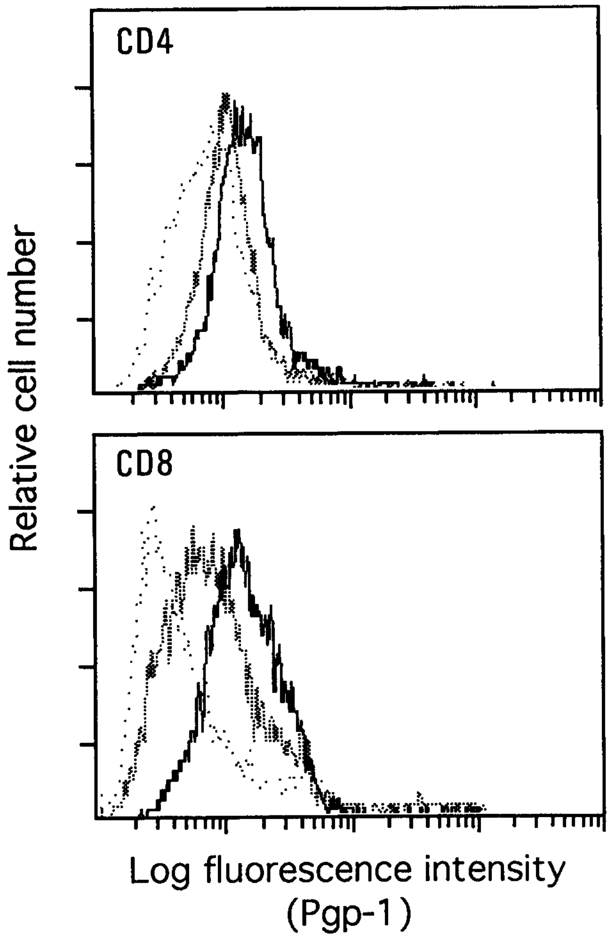

FIGURE 4. Expression of Pgp-1 on CD4 and CD8 pLN cells of 10-week-old BALB/c, C57BL/6, and NOD mice. The pLN cells were stained with FITC-anti Pgp-1 and either PE-anti-CD4 or anti-CD8. The CD4 and CD8 LN cells from C57BL/6 (dotted curves) BALB/c (solid curves), and NOD (condensed dotted curves) mice were gated and analyzed for Pgp-1 expression.

### DISCUSSION

The experiments reported herein are the first to examine the migratory properties of NOD mouse lymphocytes and to compare these properties between several mouse strains. Our results show strain-related differences in the homing of CD4 and CD8 T-cell subsets to LN and PP. In NOD and BALB/c mice, CD4 T cells preferentially migrated to these organs, whereas in C57BL/6, CBA, and DBA/2 mice, CD4 and CD8 T cells localized equally well.

Radiolabeled LN cells redistributed in similar proportions in different organs of NOD and BALB/c mice. In NOD mice, lymphocyte infiltration occurs in organs such as the pancreas, salivary glands, thyroid, and lungs (Tochino, 1987), but homing of radiolabeled LN cells in these organs was not increased. It has not yet been shown whether there is continuous migration of cells in the inflamed islets during the time between onset of insulitis and beta-cell destruction. However, we have evidence that lymphocytes still enter the pancreas late in life, suggesting that infiltrating cells recirculate (manuscript in preparation). This indicates that the number of cells that home to the islets within 2 hr is too low to be detected with radiolabeled cells.

The relative localization of FITC-labeled B and T lymphocytes was not different in NOD, BALB/c, or C57BL/6 mice. As previously shown in different strains of mice and rats (Stevens et al., 1982; Westermann et al., 1989), B cells preferentially recirculated through the spleen and PP, whereas T cells preferentially homed to LN. This selectivity is attributable to complementary lymphocyte–endothelial-cell receptor sets (Butcher et al., 1980).

In addition to tissue-specific T and B lymphocyte migration, T-cell subset-specific interactions with endothelium were observed. There is general agreement about these interactions in rats and sheep (Washington et al., 1988; Westermann et al., 1989; Abernethy et al., 1990). However, reports regarding the relative migratory properties of phenotypically defined subpopulations of T cells in mice appear controversial. Initially, the preferential migration of mouse CD4 cells was first reported for PP only (Kraal et al., 1983a). In these studies, using C57BL/6 and AKR mice, CD4 and CD8 cells localized roughly equivalently in LN. More recently, Fisher and Ottaway (1991) showed that, on the contrary, in BALB/c, CD4 cells enter LN at a much faster rate than CD8 cells. Their results, apparently divergent from those of Kraal and colleagues (1983a), in fact reflect a strain-dependent profile of homing of T-cell subsets into lymph nodes, clearly demonstrated by our results. In 10-week-old NOD and BALB/c mice, CD4 T cells preferentially migrated into LN and PP. In BALB/c mice, 3 to 5 times more CD4 than CD8 cells were found in the LN and PP. In this strain, CD4 and CD8 cells migrated with the same efficiency in the spleen. Lymphocyte homing to the spleen does not involve HEV, but the spleen has a predominant role in lymphocyte migration and thus was included in this study (Pabst, 1988). Preferential homing of CD4 cells was highest in the NOD mouse (four- to six-fold) and was observed in all organs, including the spleen. This migration pattern lasted for several weeks, hence during recirculation.

Conversely, CD4 and CD8 cells in CBA, DBA/2, and C57BL/6 mice show similar patterns of migration into LN.

The different migratory properties of CD4 and CD8 cells observed in NOD and BALB/c mice did not result from an increased blood transit time of CD8 cells. Study of the migration of the two T-cell subsets into the liver and the lungs of adult NOD mice showed preferential migration of CD8 cells in the liver (RMI = 0.5), but CD4 and CD8 cells migrated equally well in the lungs (RMI = 1).

It has been suggested that nonrandom recirculation of CD4 and CD8 T cells could be explained by subset-specific adhesion molecules whose interaction with endothelial counterreceptors would determine the rate of extraction of lymphocyte subsets of a given tissue specificity (Washington et al., 1988; Abernethy et al., 1990). On the other hand, strain-dependent differences may be related to the lymphocyte-subset representation, which is genetically controlled in inbred strains of mice (Kraal et al., 1983b). Indeed, Kraal and colleagues (1983b) reported that CD8 cells constitute a significantly greater proportion of total T cells in C57BL/6 mice than in BALB/c mice, resulting in a higher CD4/CD8 ratio in BALB/c than in C57BL/6. In this study, similar results were found. The CD4/CD8 ratio of LN cells was 1.4 ± 0.1 in C57BL/6 mice versus 2.5 ± 0.1 in BALB/c mice. In NOD mice, the CD4/CD8 ratio (2.7 ± 0.2) was identical to that in BALB/c, and the greatest differences in the relative homing of CD4 and CD8 cells were observed in these two mouse strains.

To determine whether there was a correlation between the homing characteristics of CD4 and CD8 cells and the severity of insulitis, which increases with time, migration of these subsets was studied in NOD and BALB/c mice of different ages. No major differences were observed between healthy adult (10 and 20 weeks) and diabetic NOD mice. However, in 2-week-old NOD, thus before insulitis develops, the migration of CD4 cells was hardly different from that of CD8 cells. On the contrary, no significant age-dependent differences were observed in BALB/c. Moreover, the RMI in the circulating blood of young and adult NOD mice were comparable, suggesting no difference in the blood transit time of CD4 and CD8 cells with the age of the mice. It would be interesting to determine whether the different migration patterns of the two T-cell subsets in 2-week-old and adult NOD mice may be associated with the pathogenesis of the disease. It is noteworthy that the development of the disease appears to depend upon cellular events taking place around weaning and that would be involved in the generation of suppressor T cells. Previous experiments showed that thymectomy at 3 weeks of age results in an increase in the incidence of diabetes, suggesting that suppressor mechanisms develop after that age (Dardenne et al., 1989). This was supported by the fact that diabetes can be transfered into nonirradiated mice from birth until about 3 weeks of age (Bendelac et al., 1987).

Lastly, no major differences in the expression of the MEL-14 antigen, LFA-1, and Pgp-1 could explain the different migratory properties of CD4 and CD8 cells in BALB/c, NOD and C57BL/6 mice. We confirm data from others showing higher expression of the MEL-14 antigen on CD8 cells than on CD4 cells in mice (Ohgama and Onoé, 1992), and a higher expression of Pgp-1 cells in BALB/c mice as compared to C57BL/6 mice (Lynch and Ceredig, 1989). NOD mice expressed an intermediate Pgp-1 phenotype. The regulatory mechanisms involved in Pgp-1 gene expression in mice remains unclear. Most of the cells expressed the integrin LFA-1, with a higher expression on CD8 than on CD4 cells, as for the MEL-14 antigen. The functional significance of high expression of the MEL-14 antigen and LFA-1 on CD8 cells is still unknown.

The principal conclusion arising from this study is that whether differential migration of CD4 and CD8 T-cell subsets into PP is a common feature of all mouse strains, there is strain-dependent differences in the redistribution of T-cell subsets in LN. Two groups of strains were defined. In the first group, including C57BL/6, DBA/2, and CBA, CD4 and CD8 cells migrated equally well into these organs. In the second group, comprising BALB/c and NOD mice, preferential homing of CD4 cells was observed, which was age-dependent in NOD mice but not in BALB/c mice. Thus, lymphocyte homing is controlled by strain-, organ-, and subset-specific mechanisms.

### MATERIALS AND METHODS

### Animals

NOD, CBA, and C57BL/6 mice were bred in our own facilities in specific-pathogen-free conditions. DBA/2 and BALB/c mice were purchased from Iffa-Credo (L'Arbresle, France). NOD Thy-1.1 mice were generously given by Dr. E.H. Leiter (Jackson Laboratory, Bar Harbor, ME). NOD mice were used at ages ranging from 2 to 25 weeks. BALB/c were used at 2 and 10 weeks of age and DBA/2, C57BL/6, CBA, and NOD Thy-1.1 at 10 weeks. Diabetic mice were used within the week following disease onset.

### Preparation of Cell Suspensions

Single-cell suspensions of lymphoid organs were prepared using a homogeneizer in MEM medium (containing 5 mM sodium azide for immunofluorescence staining). The cells were washed, resuspended in the same medium, filtered through a 40-$\mu$m nylon screen, and counted. For isolation of lymphocytes from the lungs and the liver, mice were perfused by the heart with PBS plus 10 U/ml heparin. Lungs and liver were collected, pressed through a 40 $\mu$m nylon screen, and suspended in MEM supplemented with 10% FCS (Watanabe et al., 1992). The suspensions were centrifuged at 1200 rpm for 5 min and the pellets were resuspended in the medium (10 ml/2 lungs and 20 ml/liver). Mononuclear cells were isolated by Ficoll density-gradient centrifugation. Cell viability was determined by the Trypan blue exclusion test.

### *In Vivo* Transfer Experiments of $^{51}$Cr-labeled Cells

Cell suspensions of pLN were prepared. $2.5 \times 10^7$ cells in 1 ml were incubated with 100 $\mu$Ci of sodium chromate ($Na_2{}^{51}CrO_4$ [CEA, France], 100 to 1,000 mCi/mg) in MEM medium for 45 min at 37°C with occasional shaking. Labeled cells were washed three times to remove free chromium. Each recipient received i.v. $3 \times 10^6$ cells in 0.2 ml of MEM medium. Two hours later, mice were perfused and different tissues were collected. Radioactivity was counted in a gamma scintillation counter. Results for each tissue are expressed as percent migration, calculated on the basis of total counts recovered in all tissues harvested.

### Antibodies

Anti-CD4 (YTS 191.1, Rat IgG2b), anti-CD8 (YTS 169.4 rat IgG2b), and anti-B220 (RA3-6B2, rat IgG2a) (Coffman, 1982) were purchased as phycoerythrin (PE)-conjugates from Caltag (San Francisco). Monoclonal antibodies to LFA-1 (H35-89.9, rat IgG) (Pierres et al., 1982), Pgp-1 (IM7, rat IgG2b) (Trowbridge et al., 1982), and the lymphocyte homing receptor (MEL-14, rat IgG2a) were obtained from ascites, purified on a protein G column, and coupled to biotin or FITC. FITC-conjugated streptavidin second stage (Amersham, Les Ulis, France) was used when appropriate.

### Immunofluorescence Staining

One million cells were pelleted in each well of microtiter plates and resuspended in 20 $\mu$l of medium, PE-conjugated reagents, or biotin-labeled antibodies at an appropriate concentration. After a 30-min incubation on ice, cells were washed twice. FITC-streptavidin was added when needed and plates were incubated for 30 min on ice. After washing, cells were finally resuspended in PBS containing 1% formaldehyde. For double staining, a mixture of two antibodies at optimal concentrations was used as first-stage antibodies, one being conjugated to PE and the other biotinylated. FITC-streptavidin was then used as mentioned before. For staining of blood cells, 50 $\mu$l of blood were incubated with 10 $\mu$l of PE-conjugated antibodies in conical tubes for 20 min on ice. Two milliliters of an RBC lysing solution (Becton Dickinson, Grenoble) were added for 5 min. The cells were then washed twice and resuspended in PBS plus 1% formaldehyde.

### *In Vitro* FITC Cell Labeling

The standard labeling procedures have been previously described (Butcher and Weissman, 1980). Briefly, $2.5 \times 10^7$ cells/ml were labeled in RPMI containing 2% FCS for 15 min at 37°C with 23 $\mu$g/ml FITC. At the end of the incubation, the labeled cells were separated from free fluorochrome by centrifugation through a 1-cm cushion of FCS.

### *In Vivo* Homing Studies

The experimental protocol described by Stevens and colleagues (1982) was used. Sample populations of mesenteric lymph nodes (mLN) and pLN (axillary and inguinal nodes) cells were pooled from nine 10- or 25-week-old donors or from thirty 2-week-old donors. Two to $2.5 \times 10^7$ cells of each population were labeled *in vitro* with FITC and injected into each of three syngeneic recipients. The recipients were killed 2 hr after injection. Cell suspensions from pLN, mLN, pancreatic LN (paLN), PP, and spleen were pooled from triplicate recipients. Organ cells from both injected animals and recipients were stained with PE-conjugated anti-CD4, anti-CD8, and anti-B220 antibodies to determine the respective proportion of each lymphocyte subset constituting both the inoculum and the migrant cells. Cells were analyzed using a FACScan (Becton-Dickinson, Grenoble). At least 500 FITC+ cells were scored per sample.

In a time course experiment, Thy-1.1 congenic recipients received i.v. $2.5 \times 10^7$ pLN cells from Thy-1.2 donors. At various time points, the recipients were killed and cell suspensions of different lymphoid organs from two recipients were pooled. These cell suspensions, as well as the original injected sample population, were stained with a mixture of FITC-anti-Thy-1.2 and PE-conjugated anti-CD4 or anti-CD8. At least 500 donor cells (Thy-1.2) were analyzed using a FACScan. The percentage of donor-type CD4 and CD8 lymphocytes was compared to the percentage of the same lymphocyte subset within the inoculum.

### Determination of the Relative Migration Index (RMI)

The CD4/CD8 ratio of donor cells was determined before injection and 2 hr posttransfer in each organ of recipients of FITC cells or Thy-1.2 cells. A relative migration index (RMI) was calculated:

$$\text{RMI} = \frac{\text{CD4/CD8 FITC-labeled cells in each recipient organ}}{\text{CD4/CD8 injected cells}}$$

Thus, an RMI greater than 1 indicates that CD4 cells migrate into the recipient lymphoid organ significantly better than CD8 cells, and vice versa.

### Expression of Adhesion Molecules in Peripheral Lymphoid Organs

Expression of MEL-14 antigen, LFA-1, and Pgp-1 by pLN of 3-, 10-, and 20-week-old NOD, BALB/c, and C57BL/6 mice was analyzed. Double staining was used to determine the percentage of cells expressing these adhesion molecules in CD4-, CD8- and B-cell subpopulations.

### ACKNOWLEDGMENTS

This work was supported by centre national de la recherche scientifique (CNRS). The authors wish to thank Mrs. M. Calise for managing the NOD colony, C. Slama for typing the manuscript, and Mrs. D. Broneer for reading the manuscript.

*(Received July 26, 1993)*

*(Accepted November 8, 1993)*

### REFERENCES

Abernethy N.J., Hay J.B., Kimpton W.G., Washington E.A., and Cahill R.N.P. (1990). Non-random recirculation of small, CD4+ and CD8+ T lymphocytes in sheep: Evidence for lymphocyte subset-specific lymphocyte-endothelia cell recognition. Int. Immunol. **2:** 231–238.

Bendelac A., Carnaud C., Boitard C., and Bach J.F. (1987). Syngeneic transfer of autoimmune diabetes from diabetic NOD mice to healthy neonates. Requirement for both L3T4+ and Lyt-2+ T cells. J. Exp. Med. **166:** 823–832.

Berg E.L., Golstein L.A., Jutila M.A., Nakache M., Picker L.J., Streeter P.R., Wu N.W., Zhou D., and Butcher E.C. (1989). Homing receptors and vascular addressins: Cell adhesion molecules that direct lymphocyte traffic. Immunol. Rev. **108:** 5–18.

Budd R.C., Cerottini J.C., Horvath C., Bron C., Pedrazzini T., Howe R.C., and Macdonald H.R. (1987). Distinction of virgin and memory T lymphocytes. Stable acquisition of the Pgp-1 glycoprotein concomitant with antigenic stimulation. J. Immunol. **138:** 3120–3129.

Butcher E.C. (1991). Leucocyte-endothelial cell recognition: Three (or more) steps to specificity and diversity. Cell **67:** 1033–1036.

Butcher E.C., Scollay R.G., and Weissman I.L. (1980). Organ specificity of lymphocyte migration: Mediation by highly selective lymphocyte interaction with organ-specific determinant on high endothelial venules. Eur. J. Immunol. **10:** 556–561.

Butcher E.C., and Weissman I.L. (1980). Direct fluorescent labelling of cells with fluorescein or rhodamine isothiocyanate. I. Technical aspects. J. Immunol. Methods **37:** 97–108.

Cannella B., Cross A.H., and Raine C.S. (1991). Relapsing autoimmune demyelination: A role for vascular addressins. J. Neuroimmunol. **35:** 295–300.

Coffman R.L. (1982). Surface antigen expression and immunoglobulin gene rearrangement during mouse pre B cell development. Immunol. Rev. **69:** 5–22.

Dardenne M., Lepault F., Bendelac A., and Bach J.F. (1989). Acceleration of the onset of diabetes in NOD mice by thymectomy at weaning. Eur. J. Immunol. **19:** 889–895.

Dinther-Janssen A.C.H.M. Van, Pals S.T., Scheper R.J., Breedveld F., and Meijer C.J.L.M. (1990). Dendritic cells and high endothelial venules in the rheumatoid synovial membrane. J. Rheumatol. **17:** 11–16

Fischer L.O., and Ottaway C.A. (1991). The kinetics of migration of murine CD4 and CD8 lymphocytes in vivo. Reg. Immunol. **3:** 156–162.

Gallatin W.M., Weissman I.L., and Butcher E.C. (1983). A cell surface molecule involved in organ-specific homing of lymphocytes. Nature **304:** 30–34.

Hamann A., Jablonskli-Westrich D., Duijvestijm A., Butcher E.C., Baisch H., Harder R., and Tiele H. (1988). Evidence for an accessory role of LFA-1 in lymphocyte-high endothelium interaction during homing. J. Immunol. **140:** 693–699.

Holzmann B., McIntyre B.W., and Weissman I.L. (1989). Identification of a murine Peyer's patch-specific lymphocyte homing as an integrin molecule with an alpha chain homologous to human VLA-A alpha. Cell **56:** 37.

Holzmann B., and Weissman I.L. (1989). Integrin molecules involved in lymphocyte homing to Peyer's patches. Immunol. Rev. **108:** 45–61.

Jalkanen S., Bargatze R.F., de Los Togos J., and Butcher E.C. (1987). Lymphocyte recognition of high endothelium: Antibodies to distinct epitopes of an 85-95-kD glycoprotein in antigen differentially inhibit lymphocyte binding to lymph node, mucosal, or synovial endothelial cells. J. Cell. Biol. **105:** 983.

Jalkanen S., Steere A.C., Fox R.O., and Butcher E.C. (1986). A distinct endothelial cell recognition system that controls lymphocyte traffic into inflamed synovium. Science **233:** 556–558.

Kraal G., Weissman I.L., and Butcher E.C. (1983a). Differences in *in vivo* distribution and homing of T cell subsets to mucosal vs non mucosal lymphoid organs. J. Immunol. **130:** 1097–1102.

Kraal G., Weissman I.L., and Butcher E.C. (1983b). Genetic control of T-cell subset representation in inbred mice. Immunogenetics **18:** 585–592.

Lynch F., and Ceredig R. (1989). Mouse strain variation in Ly-24 (Pgb-1) expression by peripheral T cells and thymocytes: Implications for T cell differentiation. Eur. J. Immunol. **19:** 223–229.

Makino S., Kunimoto K., Muraoka Y., Mizuishima Y., Katagiri K., and Tochino Y. (1980). Breeding of a non-obese diabetic strain of mice. J. Exp. Med. **29:** 1–13.

Miyazaki A., Hanafusa T., Yamada K., Miyagawa J., Fujino-Kurihara H., Nakajima H., Nonaka K., and Tarui S. (1985). Predominance of T lymphocytes in pancreatic islets and spleen of pre-diabetic non-obese diabetic (NOD) mice: A longitudinal study. Clin. Exp. Immunol. **60:** 622–630.

Mori Y., Suko M., Matsuba I., and Tsuroka A. (1986). Preventive effects of cyclosporin on diabetes in NOD mice. Diabetologia **29:** 244–247.

Ogawa M., Maruyama T., Hasegawa T., Kanaya T., Koboyashi F., Tochino Y., and Uda H. (1985). The inhibitory effect of neonatal thymectomy on the incidence of insulitis in non-obese diabetic (NOD) mice. Biomed. Res. **6:** 103–105.

Ohgama J., and Onoé K. (1992). Quantitative analysis of MEL-14 expression on various lymphocyte subpopulations. Immunobiology **186:** 268–281.

Pabst R. (1988). The spleen in lymphocyte migration. Immunol. Today **9:** 43–45.

Pals S.T., Kraal G., Horst E., de Groot A., Scheper R.J., and Meijer C.J.L.M. (1986). Human lymphocyte-high endothelial venule interaction: Organ selective binding of T and B lymphocyte populations to high endothelium. J. Immunol. **137:** 760–763.

Picker L.J., de Los Toyos J., Telen M.J., Haynes B.F., and Butcher E.C. (1989). Monoclonal antibodies against the CD44 [In(Lu)-related p80] and Pgp-1 antigens in man recognize the Hermes class of lymphocyte homing receptors. J. Immunol. **142:** 2046–2051.

Pierres M., Goridis C., and Golstein P. (1982). Inhibition of murine T cell mediated cytolysis and T cell proliferation by a rat monoclonal antibody immunoprecipitating two lymphoid cell surfacepolypeptides of 94,000 and 180,000 molecular weight. Eur. J. Immunol. **12:** 60–69.

Schizuru J.A., Taylor-Edwards C., Banks B.A., Gregory A.K., and Fathman C.G. (1988). Immunotherapy of the nonobese diabetic mouse treatment with an antibody to T-helper lymphocytes. Science **240:** 659–662.

Sempe P., Bedossa P., Richard M.F., Villa M.C., Bach J.F., and Boitard C. (1991). Anti-alpha/beta T cell receptor monoclonal antibody provides an efficient therapy for autoimmune diabetes in nonobese diabetic (NOD) mice. Eur. J. Immunol. **21:** 1163–1169.

Shimisu Y., Newman W., Tanaka Y., and Shaw S. (1992). Lymphocyte interactions with endothelial cells. Immunol. Today **13:** 106.

Springer T.A. (1990). Adhesion receptors of the immune system. Nature **346:** 425–434.

Stevens S.K., Weissman I.L., and Butcher E.C. (1982). Difference in the migration of B and T lymphocytes: Organ-selective localization *in vivo* and the role of lymphocyte–endothelial cell recognition. J. Immunol. **128:** 844–851.

Tochino Y. (1987). The NOD mouse as a model of type I diabetes. CRC Crit. Rev. Immunol **8:** 49–81.

Trowbridge I.S., Lesley J., Schulte R., Hyman R., and Trotter J. (1982). Biochemical characterization and cellular distribution of a polymorphic, murine cell-surface glycoprotein expressed on lymphoid tissues. Immunogenetics **15:** 299–312.

Washington E.A., Kimpton W.G., and Cahill R.N.P. (1988). CD4+ lymphocytes are extracted by peripheral lymph nodes at different rates from other T cell subsets and B cells, Eur. J. Immunol. **18:** 2093–2096.

Watanabe H., Ohtsuka K., Kimura M., Ikarashi Y., Ohmori K., Kusumi A., Ohteki T., Seki S., and Abo T. (1992). Details of an isolation method for hepatic lymphocytes in mice. J. Immunol. Methods **146:** 145–150.

Westermann J., Willfuhr K.U., Roth Lotter H.J., Fritz F.J., and Pabst R. (1989). Migration pattern of lymphocyte subsets in the normal rat and the influence of splenic tissue. Scand. J. Immunol. **29:** 193–201.

